# The way to a man’s heart is through his stomach?: a mixed methods study on causal mechanisms through which cash and in-kind food transfers decreased intimate partner violence

**DOI:** 10.1186/s12889-016-3129-3

**Published:** 2016-06-08

**Authors:** Ana Maria Buller, Melissa Hidrobo, Amber Peterman, Lori Heise

**Affiliations:** London School of Hygiene and Tropical Medicine, 15-17 Tavistock Place, London, WC1H 9SH UK; International Food Policy Research Institute, Washington, DC USA; UNICEF Office of Research—Innocenti, Florence, Italy

**Keywords:** Intimate partner violence, Domestic violence, Cash and in-kind transfers, Mixed methods, Ecuador, Impact evaluation, Social protection interventions

## Abstract

**Background:**

Intimate partner violence (IPV) is highly prevalent and has detrimental effects on the physical and mental health of women across the world. Despite emerging evidence on the impacts of cash transfers on intimate partner violence, the pathways through which reductions in violence occur remain under-explored. A randomised controlled trial of a cash and in-kind food transfer programme on the northern border of Ecuador showed that transfers reduced physical or sexual violence by 30 %. This mixed methods study aimed to understand the pathways that led to this reduction.

**Methods:**

We conducted a mixed methods study that combined secondary analysis from a randomised controlled trial relating to the impact of a transfer programme on IPV with in-depth interviews and focus group discussions with male and female beneficiaries. A sequential analysis strategy was followed, whereby qualitative results guided the choice of variables for the quantitative analysis and qualitative insights were used to help interpret the quantitative findings.

**Results:**

We found qualitative and quantitative evidence that the intervention led to reductions in IPV through three pathways operating at the couple, household and individual level: i) reduced day-to-day conflict and stress in the couple; ii) improved household well-being and happiness; and iii) increased women’s decision making, self-confidence and freedom of movement. We found little evidence that any type of IPV increased as a result of the transfers.

**Discussion:**

While cash and in-kind transfers can be important programmatic tools for decreasing IPV, the positive effects observed in this study seem to depend on circumstances that may not exist in all settings or programmes, such as the inclusion of a training component. Moreover, the programme built upon rather than challenged traditional gender roles by targeting women as transfer beneficiaries and framing the intervention under the umbrella of food security and nutrition – domains traditionally ascribed to women.

**Conclusions:**

Transfers destined for food consumption combined with nutrition training reduced IPV among marginalised households in northern Ecuador. Evidence suggests that these reductions were realised by decreasing stress and conflict, improving household well-being, and enhancing women’s decision making, self-confidence and freedom of movement.

**Trial registration:**

ClinicalTrials.gov NCT02526147. Registered 24 August 2015.

## Background

International data indicate that one in three women globally have experienced physical and/or sexual violence by an intimate partner (intimate partner violence or IPV) within their lifetime [1], with detrimental and long-lasting effects on their physical [2] and mental health [3]. The distribution of lifetime IPV prevalence by region varies widely, from 16.3 % of ever-partnered women aged 15 or over in East Asia to 65.64 % in central sub-Saharan Africa. In Ecuador, the setting of this study, lifetime prevalence of IPV was estimated at 35 % for physical violence, 14.5 % for sexual violence, and 43.4 % for psychological violence in the most recent national survey [4]. According to national surveys, lifetime IPV prevalence in neighbouring Colombia is also high, with 33.2 % of ever-partnered women experiencing physical partner violence and 9.7 % experiencing sexual partner violence. This suggests that Colombian refugees residing within Ecuador are likely also to be at high risk of victimisation [5, 6]. Identifying interventions that work to prevent IPV is critical to women’s health and well-being in the region and globally.

In the past two decades, cash transfer programmes in the form of cash payments to households and/or individuals have become a widely used social protection tool for decreasing household poverty and food insecurity, and for improving human capital and health. These programmes have been both piloted and implemented on a national scale across Latin America, sub-Saharan Africa and Asia, reaching up to 1 billion people in the developing world [7]. By transferring money primarily to women, these programmes often change the relative economic resource allocation between men and women in the household, potentially altering household dynamics and increasing women’s bargaining power. Existing research suggests that the relationship between cash transfers and other economic empowerment measures such as microcredit and IPV is complex and may vary by type of violence or by other contextual factors, such as the initial power dynamics between partners [8–11].

From a theoretical perspective, different paradigms lead to different hypotheses about how transfers may affect a woman’s risk of IPV. Some theories predict that an increase in resources, especially if targeted to women, may put a woman at risk of increased IPV. In this context a man may feel threatened and use violence to reassert his authority in the relationship [12, 13]. Cash and other transfers targeted at women may also put them at risk if violence is used by men to extract those resources from the women [14]. Alternatively, marital dependency and feminist theories assert that women who are economically dependent on their partner and are surrounded by institutions that promote hegemonic masculinity and gender inequality may be more susceptible to violence [15]. Furthermore, economists postulate models in which an increase in a woman’s income may decrease violence by improving her bargaining power within the household [16]. Finally, absolute resource theory and stress theory depart from bargaining or empowerment models and assert that transfers may lead to decreases in IPV by improving a household’s economic situation, thereby reducing poverty-related stressors on couples and households [15, 17].

Although there is a growing literature on the impacts of transfers on household dynamics and IPV [8, 9], few evaluations have examined the pathways through which transfers impact on IPV. To address this gap, we used a mixed methods research design to examine the impact of a cash, food and food voucher programme run by the World Food Programme (WFP) on the household dynamics and IPV risk of women in northern Ecuador. The original impact evaluation, designed as a cluster randomised controlled trial (RCT), found that overall transfers targeted at women reduced controlling behaviours and physical or sexual violence by 19 % to 30 % compared with end-line averages in control communities [18]. In this study, we draw upon the advantages of both qualitative and quantitative analysis to better understand the factors that led to this decrease. We utilise in-depth interviews (IDIs) with women and focus group discussions (FGDs) with both men and women, together with additional analysis of the quantitative data, to investigate potential pathways through which the transfer programme had an impact on IPV. Understanding how cash transfers may impact on IPV provides important programmatic implications for designing transfer programmes that achieve lasting improvements in the well-being of households and women.

## Methods

### Intervention

**Fig. 1:**
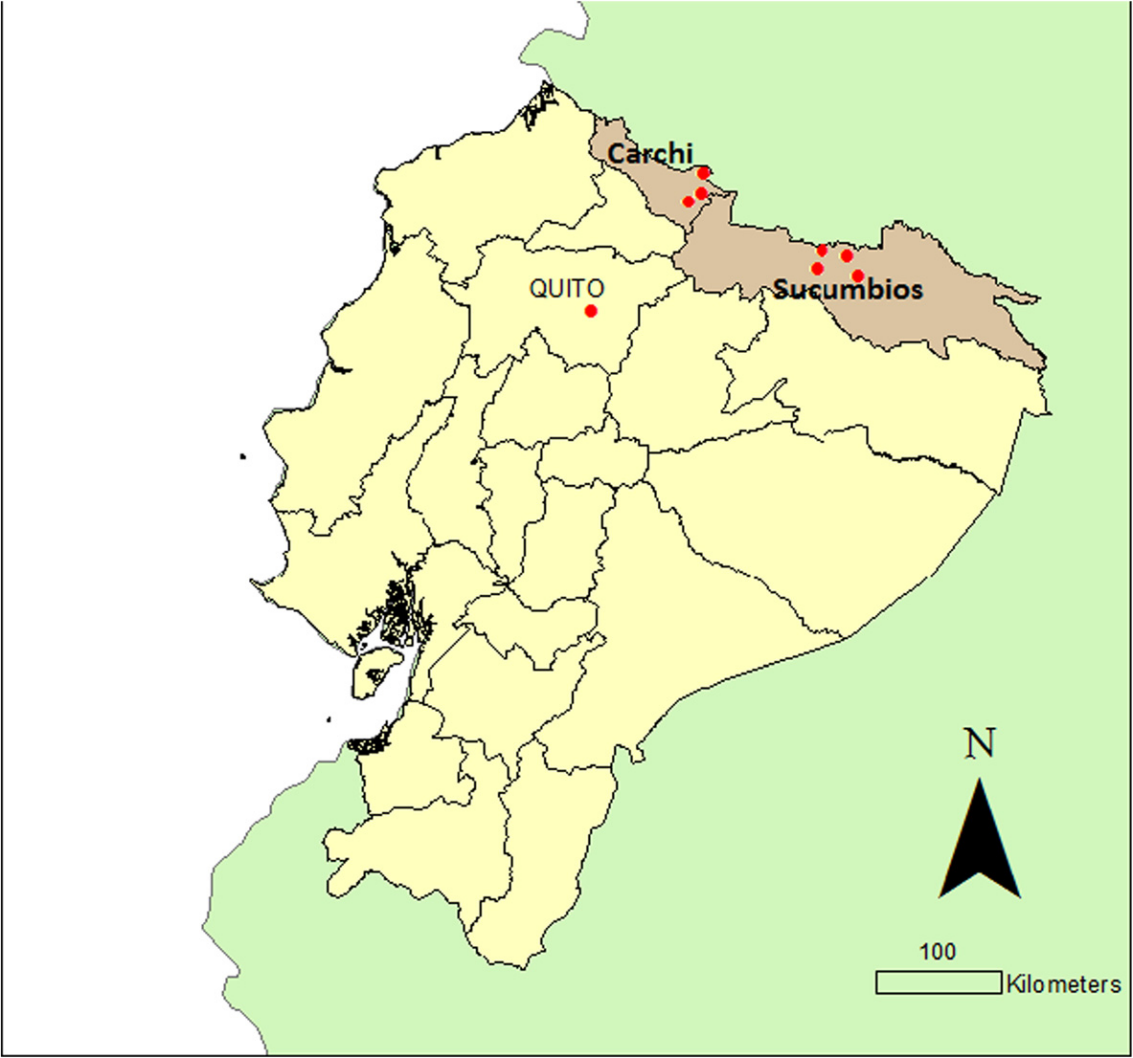
Map of intervention provinces and urban centres in Carchi and Sucumbíos

In 2011, the WFP implemented a six-month food assistance programme in seven urban centres in the northern provinces of Carchi and Sucumbíos to assist Colombian refugee and poor Ecuadorian households (Fig. 1). To determine programme qualification, all households within the selected neighbourhoods were mapped and surveyed using a short census. Households were ranked according to a poverty-focused proxy means test and a cut-off score was established – based on project budget constraints – to determine their eligibility to join the programme. The programme was targeted primarily at women and consisted of monthly transfers equivalent to $40 (approximately 11 % of a household’s pre-transfer monthly consumption) in cash, food vouchers or food transfers. The food transfer consisted of rice, lentils, vegetable oil and canned sardines and was distributed at local warehouses. The food voucher was redeemable at local supermarkets for an approved list of nutritious foods. The cash was distributed through pre-programmed ATM cards. The transfers were conditional on attendance at monthly nutrition workshops that focused on dietary diversity and child and family nutrition. Further details of the transfer intervention design and implementation have been published previously and can be found elsewhere [19].

### Research design

This study was designed as a mixed methods, sequential explanatory study [20], with alternating methods used at different phases of the research process [21]. Our quantitative results dictated the sampling for the qualitative data collection, with women being selected for the qualitative study based on the outcome of interest, namely whether IPV changed over the evaluation period (Table 1). We also utilised findings from the quantitative evaluation to inform the development of the subsequent IDI and FGD topic guides. In order to increase the validity of our findings, we examined both the quantitative and the qualitative evidence to triangulate the findings emerging from each method and to identify areas where results converged, diverged, or added insight to one another [22].

**Table 1:** Qualitative sample

In-depth interviews (IDIs)			Focus group discussions (FGDs)	
Tulcan	IPV trend	N (n modality)	Participant status	N
	Increased	10 (4 cash/3 food/3 voucher)	Men whose partners received the transfer	9 participants
	Decreased	10 (4 cash/4 food/2 voucher)	Men who received the transfer	9 participants
	Increased (Control)	4	Women beneficiaries	9 participants
			Control	5 participants
Total		24 IDIs		4 FGDs (32 participants)
Nueva Loja	Increased	10 (4 cash/4 food/2 voucher)	Men whose partners received the transfer	5 participants
	Decreased	10 (4 cash/4 food/2 voucher)	Men who received the transfer	6 participants
	Increased (Control)	4	Women beneficiaries	6 participants
			Control	3 participants
Total		24 IDIs		4 FGDs (20 participants)
TOTAL		48 IDIs (51 hours of audio)		8 FGDs (12 hours of audio)

Note: IPV trend refers to the change over the quantitative baseline and end-line surveys

#### Quantitative design

The RCT consisted of 148 clusters stratified by province and randomised into four intervention groups of cash, food, food vouchers, and control. Baseline and end-line surveys were administered to randomly selected households in each cluster in March 2011 (prior to the start of the programme) and in October/November 2011 (after the last transfer). In total, 2,357 households were surveyed at baseline and 2,122 at end-line, resulting in an attrition rate of approximately 10 %. Enumerators collected information on household characteristics, demographics, food security, education and health using face-to-face surveys. Analysis of the evaluation sample showed that the randomisation was successful at balancing characteristics across all intervention groups [18].

The household survey collected information on women’s status and IPV from one woman per household. Estimates of physical, sexual and emotional partner violence were collected in accordance with World Health Organization (WHO) ethical guidelines for conducting research on IPV [23], using standardised and internationally validated measures of violence based on the WHO Violence Against Women Instrument [24] and the Conflict Tactics Scales (CTS) [25, 26]. Because the transfers were made over a period of six months, the survey collected data on both lifetime IPV and IPV experienced over the past six months. IPV questions in the household surveys were addressed only to women aged 15 years and older who were married or partnered at the time of the interview and where privacy could be assured. Thus, of the 2,357 households at baseline, 1,433 included a woman aged 15 to 69 years and in a relationship; of these, 1,413 were alone at the time of the interview and thus completed the IPV module. The final sample of women for analysis consisted of 1,226 women aged 15 to 69 years in a relationship at baseline, with baseline and follow-up data on IPV. Attrition analysis demonstrated that selective attrition between the treatment and comparison arms was not a threat to the internal validity of the study with respect to IPV outcomes [18].

#### Qualitative design

The qualitative component included both IDIs with women and FGDs with women and men. IDIs were used to explore the women’s subjective experiences of the transfers and their household and relationship dynamics, as well as their experience of physical, emotional and sexual IPV. We also conducted FGDs with men to uncover the dominant narrative about how ‘men in their locale’ interpreted and reacted to the introduction of transfers. Given the group nature of FGDs, we did not explore personal experiences of IPV but sought more general information that was useful for decoding whether and how transfers may have disrupted gender relations and impacted on violence. The topic guides for both the IDIs and the FGDs were piloted before the fieldwork started in order to ensure that the language and questions were clear for the target population.

Qualitative fieldwork was conducted between August and September 2013 (21 months after the intervention) in the two primary urban centres in each province: Tulcan in Carchi and Nueva Loja in Sucumbíos. The interviewers were two women from Quito who had previous experience of conducting interviews with vulnerable populations and had worked in these geographical areas in the past. FGDs were conducted by the principal investigator, who has extensive experience of interviewing men about violence issues [27, 28]. The topic guides for the IDIs covered the following areas: i) background and social networks; ii) intra-household dynamics (division of work, decision-making process); iii) experience and views of the transfers; iv) attitudes towards IPV and experiences of IPV at the time of the transfers; and v) empowerment. This paper reports exclusively on data relevant to explaining the mechanisms through which transfers affected IPV.

A total of 48 IDIs and two FGDs were conducted with women purposively selected from the quantitative sample in each urban centre, stratified by experience of IPV and by transfer modality and based on their responses to the pre- and post-intervention survey. In addition, a total of six FGDs were conducted with men sampled from the treatment group following the same principles applied for the women. Men were stratified according to whether they or their female partners had received the transfer (one group representing each condition in both urban centres) or whether they belonged to the comparison group (Table 1). For safety reasons and in order to avoid putting women at risk of further violence, we did not interview or include in the FGDs men who were partners of women who were interviewed or who took part in FGDs. During interview training and supervision, we emphasised that interviewers needed to pay attention to verbal and non-verbal cues suggesting that the interviewee found the question confusing or unclear and to paraphrase questions when needed.

#### Ethics

Ethics approval for the RCT was obtained from the International Food Policy Research Institute (IFPRI) and the in-country data collection partner Centro de Estudios de Población y Desarrollo Social (CEPAR); details can be found elsewhere [18, 19]. Ethics approval for the qualitative study was obtained from IFPRI and the London School of Hygiene and Tropical Medicine (LSHTM). For both quantitative and qualitative data collection, all field staff were trained in accordance with the WHO ethical guidelines for conducting research on IPV [23]. Given participant concerns about signing written documents and literacy challenges, we relied on verbal consent for the qualitative data collection. Before giving consent, interviewees were given a simply worded information sheet and time to read it before deciding whether to take part in the study. To account for potential illiteracy, interviewers were instructed also to read the information sheet aloud and to make sure that participants understood all of its elements. No incentives were offered to participants to join the study.

Data collection, storage and analysis were designed to maintain participant confidentiality. Enumerators and interviewers provided all women, regardless of abuse status, with de-identified contact information for local IPV support services. These services included Comisarías de la Mujer y la Familia that are women-centred and staffed by women; these exist in each urban centre and include female police officers and social services. Women reporting ongoing abuse were offered the option to contact these support services. Interviews were conducted by both male and female enumerators in the quantitative survey, and by female interviewers only in the qualitative part of the study.

In order to protect interviewers from potential psychological distress, the field supervisor debriefed each interviewer at the end of the day and held wider team meetings once a week to discuss issues arising in the fieldwork and emotions associated with the content of the interviews. Interviewers found this space useful and often reported feelings of sadness when listening to women’s stories of poverty and, in some instances, violence. However, they also reported that many women thanked them at the end of the interviews because they had never had the chance to discuss these issues before, which made interviewers feel positive about their role in these women’s lives. A workshop was held in Quito, Ecuador with members of the WFP and local authorities in order to disseminate the study results to key stakeholders.

### Analysis

Integration of the quantitative and qualitative data occurred through triangulation of the data in which the two databases were used for analysis and comparison [20]. Thematic findings and analysis of the qualitative data guided the choice of variables and secondary quantitative analysis. A report and a presentation on the qualitative findings were prepared and presented. Quantitative indicators that related to the thematic findings from the qualitative report were then chosen. For each thematic area, quantitative and qualitative data were analysed and findings from both reported and compared.

#### Quantitative analysis

To estimate programme impacts on a series of possible mechanisms identified in the qualitative analysis, we conducted intent-to-treat (ITT) estimates using analysis of covariance (ANCOVA) modelling, controlling for province and the baseline value of our outcome variable. All analysis was conducted using Stata 13. As randomisation was successful in balancing baseline characteristics across treatment and control arms, we present unadjusted regression results. However, sensitivity analysis confirmed that results were unchanged with a set of standard demographic controls of the individual woman, her partner and household (not presented). Given that we found no differential impact by treatment modality on IPV in the RCT, we conducted the analysis of mechanisms using a pooled treatment indicator that included all three arms (cash, food and food voucher) [18]. Outcomes chosen for analysis were based on qualitative evidence of pathways of impact. The quantitative survey is somewhat limited in terms of questions on mechanisms, and thus for each theme we explored all relevant outcomes not previously analysed in companion papers. Outcomes were constructed and grouped as follows:

1. *Disputes and disagreements over household decision-making domains:* Binary indicators were constructed according to whether or not the woman had had a disagreement in the last six months for each of the eight domains detailed in Table 2. In addition, a summary index was constructed; this equalled one if the woman reported ‘yes’ to a disagreement in any of the eight domains.

**Table 2:** Impact of pooled treatment (cash, food or voucher) on disputes and disagreements in the last six months

Topic of dispute									
	Own work for pay	Children’s education	Children’s health	Own health	Daily food purchases	Bulk food purchases	Purchase of large assets	Use of family planning	Any dispute
Pooled treatment	−0.05	−0.00	0.03	−0.01	0.00	−0.01	0.00	0.00	−0.05
	(0.02)**	(0.02)	(0.02)	(0.02)	(0.02)	(0.01)	(0.01)	(0.01)	(0.03)*
Carchi	0.01	−0.01	−0.02	−0.02	0.02	−0.00	−0.00	−0.00	−0.01
	(0.02)	(0.02)	(0.01)*	(0.01)	(0.01)	(0.01)	(0.01)	(0.01)	(0.03)
*N*	1,206	910	992	1,224	1,211	1,092	1,082	931	1,226
Pseudo R2	0.02	0.01	0.01	0.01	0.01	0.00	0.01	0.04	0.01

Notes: Marginal effects reported of probit regression results

Standard errors in parenthesis clustered at the cluster level: *p < 0.1; **p < 0.05; ***p < 0.01

All regressions control for the baseline value of outcome variable and province

2. *Locus of control and happiness:* For the four questions pertaining to control, indicators were created that equalled one if the respondent agreed with the statement (on a Likert scale; Table 3). A happiness indicator was created from a question that asked: ‘Overall, how do you feel these days?’ Respondents could choose an answer from a five-point range from ‘very happy’ to ‘very unhappy.’ An indicator was created if the respondent indicated she was very happy (Table 3).

**Table 3:** Impact of pooled treatment (cash, food or voucher) on locus of control and happiness

	My life is determined by my own actions	I have the power to make important decisions that change the course of my life	I am capable of protecting my own interests	I am satisfied with my life	I am very happy
Pooled treatment	0.02	0.04	0.04	0.01	0.03
	(0.02)	(0.03)	(0.02)**	(0.03)	(0.02)
Carchi	0.04	0.05	0.02	0.06	−0.02
	(0.02)**	(0.03)**	(0.02)	(0.03)**	(0.02)
*N*	1,226	1,226	1,226	1,226	1,226
Pseudo R2	0.01	0.02	0.02	0.03	0.04

Notes: Marginal effects reported of probit regression results

Standard errors in parenthesis clustered at the cluster level: *p < 0.1; **p < 0.05; ***p < 0.01

All regressions control for the baseline value of outcome variable and province

3. *Health and nutrition knowledge:* Women were asked to respond to a module measuring the basic health and nutrition knowledge discussed in the nutrition training sessions. Binary indicators were created for correct responses to six questions on infant feeding practices, nutritious food items and safe drinking water (Table 5).

4. *Participation in groups:* A module on participation in groups and associations was used to identify whether respondents were part of social groups ranging from community and agricultural associations to cultural, religious or political groups (Table 6).

#### Qualitative analysis

Audio recordings from the IDIs and FGDs were transcribed verbatim and uploaded onto NVivo 10. Analysis followed a thematic approach using a constant comparative method. The initial coding was guided by the themes in the topic guide sections but we also allowed for new themes to emerge from the data. To ensure inter-rater reliability, initially five IDIs and two FGD transcripts were coded by two researchers and codes compared. Where necessary, the coding framework was modified to reflect the process of reconciliation. Furthermore, the triangulation of the qualitative and quantitative data conferred validity to our results. The choice of quotes to present in the results was based on their ability to reflect common views among participants. When quotes represent an exception or deviant case, we have noted this in the text [29]. When comparing the results by IPV experience status (i.e. increase or decrease in IPV), we found no differences between the groups: all of the women who reported increased violence during or after the transfers linked this escalation of violence to other contextual issues occurring within the relationship rather than to factors related to the transfer.

## Results

**Fig. 2:**
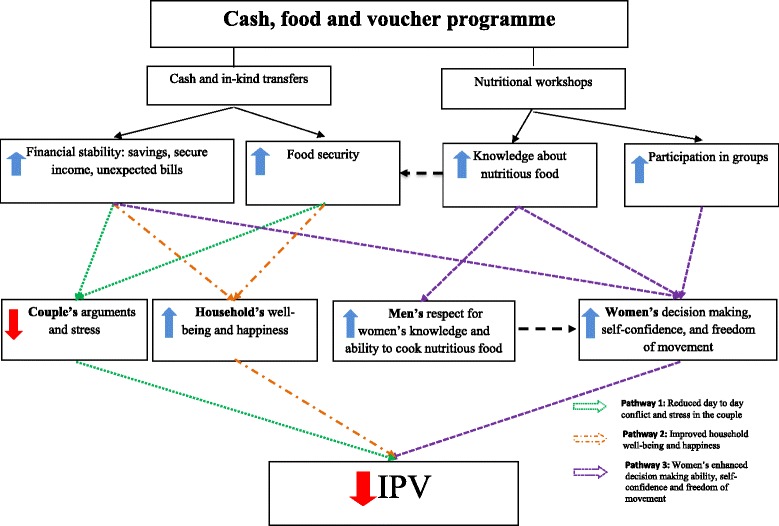
Pathways flowchart

Overall, our combined results suggest that the programme, in the form of transfers and nutrition workshops, had an impact through three sometimes overlapping protective pathways operating at the couple, household and individual level. In the first pathway, financial stability and food security combined to decrease marital conflict resulting from the daily negotiation of money to meet daily food needs. In the second pathway, financial stability and food security increased the family’s sense of well-being and happiness. Finally, in the third pathway, financial stability, knowledge about nutritious food and participation in groups had a compound effect, increasing women’s self-confidence, decision making and freedom of movement. A summary of these findings is depicted in Fig. 2 and we present a detailed explanation and evidence for each of them in the following sections. We also examined potential risk mechanisms mentioned in the literature through which transfers have the potential to increase violence. These included: i) men’s desire to extract the transfers; and ii) increased alcohol consumption by male partners, particularly as a result of increased access to cash.

### Contextual findings around IPV

The numbers of women’s individual reports of IPV in both the quantitative and qualitative studies were high. Lifetime reports of IPV in the survey showed a prevalence of 35 % for physical and/or sexual partner violence. IPV in the last six months was lower, with 26 % experiencing emotional violence and 16 % experiencing physical or sexual violence [18]. IDIs and FGDs revealed that the most common triggers of IPV were fighting over financial strains, alcohol consumption, jealousy and unfaithfulness. Men having outside sexual partners or ‘parallel families’ was a very common narrative arising from the IDIs. Usually, when women confronted their partner about infidelity, the men accused the woman of being the one who was having an affair or retaliated with violence.

### Transfers and protective pathways

#### Pathway 1: Reduced day-to-day conflict and stress in the couple

One of the main triggers of conflict in the household was the lack of financial means to fulfil basic needs such as food. In this context of extreme poverty, the norm was that women asked men for ‘*el diario*’ (the daily money) in order to purchase food for the family that day; when the man did not have enough money or refused to provide it, the resulting arguments had the potential to turn violent. Accordingly, most women mentioned that they felt stressed and had feelings of frustration and guilt about not being able to feed their children properly, and this forced them to resort to ‘*el fiado*’, a kind of informal credit established with their nearest grocery store. Thus, the elimination of the daily need to ‘negotiate’ for *el diario*, given the financial stability and food security created by the transfers, is one plausible mechanism through which transfers could have reduced IPV (Fig. 2, Pathway 1). The qualitative data suggest considerable support for this mechanism.

Interviewer: *During the time of the transfers, was there any physical violence?*

Respondent: *No, because I did not ask for it [money for food] anymore… I did not insist anymore, because the arguments happened mostly when I asked for money for food, and he usually did not have any money.* (IDI 42, Female, Tulcan)

*Sometimes problems arise because I am in need [of money] for one or the other thing and there is no money and that is when problems start, the fights […] and it [transfers] helped us a lot, he [the partner] had money to buy other things for the house or pay debts.* (IDI 35, Female, Nueva Loja)

Consistent with the qualitative results, we found that transfers led to a 5 percentage point decrease in any disputes (marginal effect = −0.05, *p* < 0.1). When looking at particular areas of potential disagreement, this appeared mainly to be due to reduced disagreements over women working for pay (marginal effect = −0.05, *p* < 0.02) (Table 2). Reports of disputes over small food purchases were low (5 %) in the quantitative survey, suggesting that they may have been under-reported, or that women did not classify the tensions over money for daily food purchases as reported in the IDIs as disputes over decision making per se, the frame used in the quantitative survey.

#### Pathway 2: Improved family well-being and happiness

Many men and women reported that the transfers brought a sense of well-being and happiness in the household. In a context where full-time, stable work is scarce, the financial stability and increased food security offered by the transfers allowed households to pay the rent, purchase important goods and services, pay debts, pay unexpected bills such as those arising from a sudden illness in the family, and ultimately save some money: ‘*the money we used to spend in food [with the transfers], we could save that and invest it in other things*’ (IDI 40, Female, Tulcan). Consequently, overall satisfaction increased, improving family relationships and well-being:

*In my household it was like happiness, we all got along, with my children, with my husband […] in my house we were happy […] because before we did not have enough money for those things [food].* (FGD 4, Female, Tulcan)

The increased food security had a direct impact on children, who, according to their parents, felt freer to request foods that they liked such as yoghurt, a source of calcium encouraged in the nutrition training workshops. Shopping for food also became a household activity that many beneficiaries looked forward to: ‘*We used to go together as a family to the supermarket and ate [after that], that was very nice, it seems to be something that is not necessary but it does contribute towards household harmony*’ (IDI 36, Female, Tulcan). Consistent with the qualitative findings, the RCT found that the transfers led to improved financial stability and food security as measured by the household’s total value of consumption, the value of food consumption, caloric intake and dietary diversity [19]. Additionally, Table 3 shows that transfers significantly increased the probability of an individual feeling capable of protecting his or her own interests – the increase here was 4 percentage points (marginal effect = 0.04, *p* < 0.05) – but had no impact on other locus of control and happiness indicators. When comparing the impact of the transfers on households in the two lowest food consumption quartiles with those in the two highest, we found large and significant improvements (from 5 to 7 percentage points) in happiness and locus of control among the poorer households (marginal effects = 0.05 to 0.07, *p* < 0.1 to *p* < 0.01) (Table 4). Thus, improved happiness and satisfaction may have been a particularly relevant way in which transfers affected IPV in the poorer households (Fig. 2, Pathway 2).

**Table 4:** Impact of pooled treatment (cash, food or voucher) on locus of control and happiness by food security quartiles

	Bottom two quartiles					Top two quartiles				
	My life is determined by my own actions	I have the power to make important decisions that change the course of my life	I am capable of protecting my own interests	I am satisfied with my life	I am very happy	My life is determined by my own actions	I have the power to make important decisions that change the course of my life	I am capable of protecting my own interests	I am satisfied with my life	I am very happy
Pooled treatment	0.05	0.07	0.05	−0.00	0.06	−0.01	−0.00	0.03	0.02	−0.00
	(0.02)**	(0.04)*	(0.03)*	(0.04)	(0.02)***	(0.03)	(0.03)	(0.02)	(0.03)	(0.03)
Carchi	0.09	0.08	0.02	0.03	−0.05	0.00	0.02	0.03	0.09	0.02
	(0.03)***	(0.04)**	(0.03)	(0.04)	(0.02)**	(0.03)	(0.03)	(0.02)	(0.03)***	(0.03)
*N*	656	656	656	656	656	570	570	570	570	570
Pseudo R2	0.04	0.03	0.02	0.04	0.09	0.00	0.01	0.02	0.02	0.02

Notes: Marginal effects reported of probit regression results

Standard errors in parenthesis clustered at the cluster level: *p < 0.1; **p < 0.05; ***p < 0.01

All regressions control for the baseline value of outcome variable and province

#### Pathway 3: Women’s decision-making ability, self-confidence and freedom of movement

The third pathway through which transfers may have decreased IPV is by empowering female beneficiaries, and in particular by increasing their ability to make decisions, their self-confidence and their freedom of movement. These changes could be related to their improved financial situation, their increased nutritional knowledge, and/or their increased participation in social groups through attendance at nutrition workshops. Qualitative interviews revealed an increase in the ability of women to make decisions, with some women mentioning that during the time of the transfers they were also ‘head of the household’: ‘*when I got that [the transfer] it was both of us [head of the household] because with what I got [the transfer] I could buy food and all and he could pay for other things*’ (IDI 31, Female, Tulcan). The interviews, however, also revealed that once the transfers ended, their increased decision-making ability diminished and they again depended on their husbands.

In contrast to the qualitative findings, quantitative findings did not clearly demonstrate that the transfers had an impact on the proportion of women with either a sole or joint decision-making role on any of the discrete decision domains or on any composite index of decision making [30]. This could be because the questions were not specific enough or sufficiently nuanced to reflect the type of enhanced influence that women expressed as deriving from the transfers. The qualitative component offered an alternative explanation for this discrepancy. When asking women in more depth about the decision-making process, we found that by ‘joint decision making’ they meant that they always asked their spouse or partner so that they would not be blamed if something went wrong. They also mentioned that men were ultimately in charge of taking the important decisions because ‘*the man is always the head of the household*’ (FGD 5, Male, Nueva Loja). These ambiguities suggest that there is room for improvement in methodologies for measuring individual intra-household economic empowerment.

Another important empowering factor may have been increased self-confidence related to women’s attendance at the monthly nutrition training sessions (Fig. 2, Pathway 3). As a woman observed: ‘*Because it [attendance at nutrition workshops] really helped me to know how to feed my family*’ (IDI 9, Female, Nueva Loja). Men also seemed to appreciate this newly gained knowledge, which improved the family’s quality of life. When asked about the impact of the transfers on their relationship, many men mentioned enhanced cooking abilities and nutritional knowledge as factors that made them feel more in love with their partners, which corresponds to the traditional role of women as those responsible for the family’s nutrition:

*Well, I think that it [relationship with a partner] improved a lot, because as we were saying, the way to a man’s heart is through his stomach, so the basic food improves the relationship and the family gets more integrated*. (FGD 6, Male, Nueva Loja)

*As I was telling you, the woman knows about her food and in my case, my wife is very good at seasoning, and you fall more in love depending on how the woman cooks… nutritiously and all*. (FGD 1, Male, Tulcan)

In order to triangulate these findings, we investigated whether the transfer programme increased women’s knowledge about health and nutrition. Programme participation showed significant impacts on several key knowledge outcomes, particularly related to food sources of iron (marginal effect = 0.13, *p* < 0.01) and vitamin A (marginal effect = 0.15, *p* < 0.01); however, there were no impacts on knowledge about infant feeding or methods of making water safe for drinking (Table 5). Women had high baseline knowledge on these latter indicators, which may partially explain why there was little improvement or ability to detect significant increases.

**Table 5:** Impact of pooled treatment (cash, food or voucher) on health and nutritional knowledge

	Baby should start breastfeeding immediately	Complementary feeding starts at 6 months	Year-old baby should eat different foods	Can name at least one source of iron-rich food	Can name at least one source of vitamin A-rich food	Can name at least one way to safely treat water for drinking
Pooled treatment	0.02	0.04	−0.03	0.13	0.15	0.00
	(0.03)	(0.03)	(0.03)	(0.02)***	(0.03)***	(0.01)
Carchi	0.02	0.06	−0.00	−0.01	0.05	0.03
	(0.02)	(0.02)**	(0.03)	(0.02)	(0.03)	(0.01)***
*N*	1,226	1,226	1,226	1,226	1,226	1,226
Pseudo R2	0.03	0.03	0.01	0.06	0.03	0.04

Notes: Marginal effects reported of probit regression results

Standard errors in parenthesis clustered at the cluster level: *p < 0.1; **p < 0.05; ***p < 0.01

All regressions control for the baseline value of outcome variable and province

Qualitatively, women reported that they enjoyed the monthly training workshops because they gave them an opportunity to interact with other women and the trainers. For some women, the training was an opportunity to be less shy and to participate in the public domain:

*I always spend my time at home, I do not go out much, I do not know how to relate with people very well and there [in the workshops], you slowly lose the shyness […] I used to be very shy, now I am only a bit shy […] I did not use to go to talks, I had never had talks before*. (IDI 2, Female, Nueva Loja)

In addition, attending the training sessions and picking up the food transfers or vouchers presented women with an opportunity to go to an activity on their own without having to face the jealousy of their partners:

Interviewer: *And during the time of the transfers when you had to go downtown to receive the food, did he also accuse you of being with a lover?*

Respondent: *No, because I used to go with more people [who also received the transfers] and I used to come back with them, sometimes in a car and he did not use to say anything.* (IDI 6, Female, Nueva Loja)

Some women, however, mentioned that men took them to the workshops and waited outside or went with them to pick up the transfers. The quantitative data support the qualitative findings on community involvement and freedom of movement. Table 6 presents results showing the marginal effects of the transfers on women’s participation in groups and associations in the last six months, and reveals that the transfer programme significantly increased women’s participation in non-governmental organisations, educational, cultural or other groups (marginal effect = 0.13, *p* < 0.01); this may represent a knock-on effect from their participation in the nutrition workshops. As demonstrated elsewhere [18], the transfer programme also led to decreased controlling behaviours from partners, which might have increased women’s freedom of movement. In particular, the transfer programme significantly decreased the probability of a partner accusing a woman of being unfaithful and attempting to limit her contact with her family.

**Table 6:** Impact of pooled treatment (cash, food or voucher) on group participation in the last six months

	Agriculture association, union or cooperative	Religious or spiritual group	Community or neighbourhood association	Political group or movement	Non-governmental organisation, educational, cultural or other group
Pooled treatment	−0.03	−0.00	−0.00	−0.01	0.13
	(0.02)*	(0.03)	(0.04)	(0.01)	(0.04)***
Carchi	−0.03	−0.01	0.03	−0.01	0.03
	(0.02)*	(0.03)	(0.03)	(0.01)	(0.03)
*N*	1,226	1,226	1,226	1,226	1,226
Pseudo R2	0.09	0.07	0.02	0.04	0.03

Notes: Marginal effects reported of probit regression results

Standard errors in parenthesis clustered at the cluster level: *p < 0.1; **p < 0.05; ***p < 0.01

All regressions control for the baseline value of outcome variable and province

### Transfers and risk pathways

#### Violent extraction of the transfers

Although there is the potential for transfers, particularly cash, to be extracted forcefully from women, there were no qualitative reports of male partners attempting to do so or wanting to use the transfer for something other than food. On the contrary, many women mentioned that their partners ‘respected’ the transfer and were happy with it being used for nutritional purposes. A few women reported men’s jealousy over the ATM card or cash; however, despite this, men respected women’s control over the transfer:

Interviewer: *Was he, like, jealous because you received the transfers? Did he ever say something like ‘Why do you get it and not me?’*

Respondent: *Not really, never, because it [the transfer] was for the sake of the household and the family*. (IDI 37, Female, Tulcan)

This dynamic was reinforced by men who made it clear that the transfer was primarily seen as money for the household rather than the woman’s money, and thus it was perceived as non-threatening to men.

*[…] I think that if there is another opportunity [another transfer programme] women should keep receiving it […] In my case, for example, if they send me to do the shopping I would only buy noodles [laughing]; the ladies, on the other hand, know what to get, they cook and can decide […] Women are the ones that know more about these things.* (FGD 2, Male, Tulcan)

Overall, men and women felt that, regardless of who received the transfer, women would likely continue to control the money because traditional gender norms dictate that shopping and food preparation is within the ‘woman’s domain’. Most of the time it was men who worked outside the home and they would not be available to receive the transfer or shop for food. However, some men and women maintained that it was better that women received the transfers because they are generally more responsible, are more aware of food issues, and have the interest of the household at heart: ‘*Because women know what is needed in the house, but men might buy one or two things and will pocket the rest to go and play volleyball*’ (IDI 20, Female, Nueva Loja).

#### Alcohol consumption

Qualitatively, none of the interviewees mentioned increased alcohol consumption as a by-product of the transfers. However, some women mentioned that transfers increased the money their spouse or partner had to spend with their ‘other women’, which might have included spending on drinking. In the baseline and end-line surveys, the reports of household alcohol consumption were too low (1 % to 2 % of households reported consuming alcohol) to explore quantitatively the possibility that transfers increased alcohol consumption.

## Discussion

Using qualitative and quantitative triangulated methods, we explored the potential mechanisms that might explain the reduction in IPV found in the original RCT. We found that IPV is a common problem in the study setting. When exploring common triggers of IPV, we found that jealousy, financial strain and men’s alcohol consumption were most often cited; this is consistent with other studies from the region [28, 31–33]. Reducing conflicts over money and financial stress appeared to be one of the main ways in which the transfers helped reduce IPV. Specifically, by creating financial stability and increasing the household’s food security, the transfers eliminated the need for women to negotiate money for each day’s food, thus reducing arguments between women and their partners. The transfers also had an impact at the household level by increasing overall well-being and happiness, a result that seemed particularly relevant for the poorest households. In addition, qualitatively, transfers appeared to increase women’s decision-making power and self-confidence, and to enhance women’s freedom of movement by decreasing men’s controlling behaviour; these impacts were largely derived from women’s participation in the monthly nutrition training workshops, which resulted in increased knowledge and enhanced group participation.

Our findings on pathways appear most consistent with economic and gender theories of violence related to stress, women’s empowerment, and marital dependency [15, 17, 34]. These theories would predict that transfers should decrease the average likelihood of IPV. We did not find any evidence to support extraction theories that would predict that transfers, especially those provided as cash rather than as vouchers or food, might increase women’s risk of violence. It is important to note that a portion of women who received transfers still experienced IPV within the recall period; this is because of the high level of violence existing within the context of the intervention. However, none of the interviewees reporting violence after the transfers linked this escalation of violence to the transfer.

The RCT results [18] indicating a reduction in IPV were measured only over the six-month period of the transfer programme. However, a longer-term programme that continued to reduce poverty-related stress and conflict and improve women’s bargaining power would likely have similar impacts on IPV; other dynamics such as employment and marriage decisions would probably be affected in a longer-term programme as well. The qualitative findings suggest that the programme’s impacts on IPV may not have been sustained when the programme ended, with increased conflict and stress returning once the transfers ceased. The nutrition training, however, seemed to yield lasting benefits. During the qualitative interviews, women reported still remembering the knowledge gained in the training workshops; many said that this was the most helpful part of the programme and that it was still applicable, even if they were in financially difficult circumstances. Further, the respect and admiration for this knowledge and women’s improved cooking skills is a useful concept to recognise, and perhaps an underutilised facet of food security programming and research, particularly within quantitative evaluations.

Significantly, both the characteristics of the setting and the framing of the programme itself may have shaped its impact on IPV. In particular, the focus of the programme on nutrition and food security clearly aligned the transfers with existing gendered expectations in the community. Since women have always been the main individuals responsible for food purchases and food preparation, the transfer was seen as a benefit for the whole household, and one that did not challenge traditional gender roles, even though resources were being placed in the hands of women. It is possible that if the transfer had been framed differently – for example as a women’s empowerment programme – men’s responses and the resulting impact on IPV would have been different. This might help explain the seemingly contradictory findings in terms of transfers and IPV that can be found in the literature.

This framing is in line with other social protection programmes linked to children’s education or health, which typically take gender into account, but in ways that reinforce stereotypical gender roles [35–37]. Holmes and Jones [35] argue that targeting social transfers to women does not necessarily empower them and that gender-sensitive design does not always translate into gender-equitable impacts. Therefore, there is a need to think about perpetuating traditional gender roles versus challenging them, and the consequences in terms of programme equity and efficiency as well as gender dynamics within the household.

Some limitations of this study are worth mentioning. Firstly, since there was a 21-month lag between the quantitative evaluation and the qualitative data collection, it is possible that there are recall issues for men and women in remembering events and dynamics around the time of the transfer programme. Secondly, although every effort was made verbally and in writing to explain to the participants that the research team was not affiliated with the transfer implementing organisation (WFP) and that there were no plans for future transfers, some individuals may have given overly positive answers in the hope of encouraging a renewal of the transfer programme. This could have resulted in women de-emphasising any potential links between the transfers and violence. However, we would not expect these biases to be quantitatively different between the control and treatment groups, and therefore they would not affect the internal validity of the findings. On the other hand, for the qualitative results, since the intervention was framed as ‘food security and nutrition’, even if the respondents thought that the interviews and the intervention were connected, it is unlikely that they assumed that their answers to IPV-related questions would influence their eligibility for a potential future transfer. Furthermore, the triangulation of quantitative and qualitative results provided further validity to our findings. Moreover, detailed data on plausible mechanisms through which transfers might have an impact on IPV were not collected in the survey. For future research it would be advantageous to collect data related to these mechanisms, such as specific stress indicators or biomarkers [38]. Finally, since all beneficiaries received transfers in addition to nutrition training, it is impossible to quantitatively disentangle the effects of the transfer alone from those of the training.

Additionally, our qualitative data suggest that much of what is reported as ‘joint decision making’ may be driven by a desire on the woman’s part to avoid being blamed if something goes wrong rather than true partnership. There have been several critiques of quantitative indicators of decision making as a measure of women’s empowerment, primarily due to the inability of decision making to fully capture the multidimensional nature of empowerment [39, 40]. In a cross-country study, Peterman and colleagues [30] found that, despite testing various ways to measure decision making, they could find no impact of cash or in-kind transfers on decision making across countries. Therefore, we recognise the need for better tools to quantitatively measure decision making and empowerment in order to assess the impact of these programmes on women’s lives.

A number of important policy and programmatic lessons can be drawn from the results presented in this paper. First, policy makers and programme implementers need to think strategically about programme objectives and whether or not they are seeking to implement programmes that are gender transformative. This means incorporating a gender lens from the design stage of the intervention and potentially complementing transfers with activities that guarantee a deep and sustained change in gender-related attitudes and expectations [35]. In some contexts, depending on the objectives of the intervention, there should be recognition of instances when gender-neutral programming can nonetheless benefit women by affecting the structural determinants of IPV. There is also a need to go beyond establishing whether an intervention reduces IPV in order to understand how it does so, under what conditions, and whether the impacts are sustained after the programme ends. As transfers may affect decision making, stress and conflict only for the duration of the programme, economic interventions may need to be combined with complementary approaches to achieve a long-term reduction in IPV risk.

## Conclusion

Despite the caveats mentioned in the discussion section, this study shows that transfers destined for food consumption combined with nutrition training reduced IPV among marginalised households in northern Ecuador. Evidence suggests that these reductions were realised by decreasing stress and conflict, improving household well-being, and enhancing women’s decision making, self-confidence and freedom of movement.

## Abbreviations

3ie, International Initiative for Impact Evaluation; ANCOVA, analysis of covariance; CEPAR, Centro de Estudios de Población y Desarrollo Social (Centre for Population Studies and Social Development); CGIAR, Consortium of International Agricultural Research Centers; CTS, Conflict tactic scales; FGD, focus group discussion; IDI, in-depth interview; IFPRI, International Food Policy Research Institute; IPV, intimate partner violence; ITT, intent-to-treat; LSHTM, London School of Hygiene and Tropical Medicine; RCT, randomised controlled trial; WFP, World Food Programme; WHO, World Health Organisation.
